# Rhythmic Mechanisms Governing CAM Photosynthesis in *Kalanchoe fedtschenkoi*: High-Resolution Temporal Transcriptomics

**DOI:** 10.3390/ijms27031342

**Published:** 2026-01-29

**Authors:** Rongbin Hu, Sara Jawdy, Avinash Sreedasyam, Anna Lipzen, Mei Wang, Vivian Ng, Christopher Daum, Keykhosrow Keymanesh, Degao Liu, Alex Hu, Asher Pasha, Nicholas J. Provart, Anne M. Borland, Timothy J. Tschaplinski, Gerald A. Tuskan, Jeremy Schmutz, Xiaohan Yang

**Affiliations:** 1 Biosciences Division, Oak Ridge National Laboratory, Oak Ridge, TN 37831, USA; rongbin.hu@ucr.edu (R.H.); jawdys@ornl.gov (S.J.); degao.liu@ttu.edu (D.L.); tschaplinstj@ornl.gov (T.J.T.); tuskanga@ornl.gov (G.A.T.); 2 The Center for Bioenergy Innovation, Oak Ridge National Laboratory, Oak Ridge, TN 37831, USA; 3 HudsonAlpha Institute for Biotechnology, 601 Genome Way, Huntsville, AL 35801, USA; asreedasyam@hudsonalpha.org (A.S.); jschmutz@hudsonalpha.org (J.S.); 4 Department of Energy, Joint Genome Institute, Lawrence Berkeley National Laboratory, Berkeley, CA 94589, USA; alipzen@lbl.gov (A.L.); mwang@lbl.gov (M.W.); vng@lbl.gov (V.N.); cgdaum@lbl.gov (C.D.); kkeymanesh@lbl.gov (K.K.); 5 Institute of Genomics for Crop Abiotic Stress Tolerance, Department of Plant and Soil Science, Texas Tech University, Lubbock, TX 79409, USA; flyingalex.hu@gmail.com; 6 Department of Cell & Systems Biology/Centre for the Analysis of Genome Evolution and Function, University of Toronto, Toronto, ON M5S 3B2, Canada; asher.pasha@utoronto.ca (A.P.); nicholas.provart@utoronto.ca (N.J.P.); 7 School of Natural and Environmental Science, Newcastle University, Newcastle upon Tyne NE1 7RU, UK; anne.borland@newcastle.ac.uk

**Keywords:** crassulacean acid metabolism, gene expression, photosynthesis, circadian clock, stomatal movement, water-use efficiency, drought stress

## Abstract

Crassulacean acid metabolism (CAM) is a specialized photosynthetic pathway that enhances water-use efficiency by temporally separating nocturnal CO_2_ uptake from daytime decarboxylation and carbon fixation. To uncover the regulatory mechanisms coordinating these temporal dynamics, we generated high-resolution, 48 h time-course transcriptomes for the CAM model *Kalanchoe fedtschenkoi* under both 12 h/12 h light/dark (LD) cycles and continuous light (LL). A rhythmicity analysis revealed that diel light cues are the dominant driver of transcript oscillations: 16,810 genes (54.3% of annotated genes) exhibited rhythmic expression only under LD, whereas just 399 genes (1.3%) remained rhythmic under LL. A smaller set of 3009 genes (9.7%) oscillated in both conditions, indicating that the intrinsic circadian clock sustains rhythmicity for a limited subset of the transcriptome. A gene co-expression network analysis revealed extensive integration between circadian clock components, core CAM pathway enzymes, and stomatal regulators, defining regulatory modules that coordinate metabolic and physiological timing. Notably, key hub genes associated with post-translational and post-transcriptional regulation, including the E3 ubiquitin ligase HUB2 and several pentatricopeptide repeat (PPR) proteins, act as central nodes in CAM-associated networks. This discovery implicates epigenetic and organellar regulation as previously unrecognized critical tiers of control in CAM. Together, our results support a regulatory model in which CAM rhythmicity is governed by both external light/dark cues and the endogenous circadian clock through multi-level control spanning transcriptional and protein-level regulation. To support community exploration, we also provide an interactive eFP (electronic Fluorescent Pictograph) browser for visualizing time-resolved gene expression profiles.

## 1. Introduction

Crassulacean acid metabolism (CAM) is an adaptive photosynthetic pathway that enables plants to possess higher water-use efficiency (WUE) compared to C_3_ and C_4_ species, leading to enhanced tolerance to drought stress [1,2,3]. In general, CAM physiology features four phases: (1) Phase I, stomata open at night to take up atmospheric carbon dioxide (CO_2_) through a carboxylation process mediated by PHOSPHOENOLPYRUVATE CARBOXYLASE (PEPC). The CO_2_ is converted to malate and subsequently stored in the vacuole as malic acid. (2) Phase II, CO_2_ uptake shifts from PEPC to ribulose-1-5-bisphosphate carboxylase/oxygenase (RuBisCO) after dawn. (3) Phase III, during the daytime, malic acid undergoes decarboxylation to release CO_2_, which is then refixed by RuBisCO in the Calvin cycle, while the stomata remain closed to reduce water loss through evaporation. (4) Phase IV, CO_2_ undergoes direct fixation by RuBisCO with limited stomatal opening in the late afternoon [2,3].

The regulation of CAM involves the spatial and temporal compartmentalization of carbon fixation and relies on transcriptional, post-transcriptional, and post-translational modifications that are thought to be regulated by the circadian clock network [4,5,6]. In CAM species, photosynthetic efficiency is affected not only through optimized carbon assimilation but also through accurate temporal regulation of nocturnal CO_2_ uptake in concert with stomatal opening, malate storage, and daytime decarboxylation with stomatal closure. The circadian clock therefore functions as an essential temporal organizer, ensuring that metabolic fluxes, enzyme activities, and stomatal behavior occur at the appropriate phases of the diel cycle. While core circadian clock components, such as CCA1, LHY, TOC1, and the REVEILLE family, are functionally conserved across plant lineages, their downstream transcriptional reprogramming differs substantially between CAM and C_3_ species, defining the unique temporal demands of CAM metabolism [5,6,7,8,9]. Furthermore, an inverted stomatal exhibition in CAM plants suggests a unique and critical set of regulatory mechanisms in guard cell pathways, ion transport activities, and hormonal signaling, likely related to abscisic acid (ABA) and Ca^2+^ signaling, which integrate environmental cues to regulate gas exchange and WUE [10,11,12].

*Kalanchoe fedtschenkoi* (Raym.-Hamet & H. Perrier) is an obligate CAM species that has emerged as an ideal model for studying CAM regulatory mechanisms due to its relatively small size, low maintenance requirements, and amenability to transformation [2,13,14,15,16]. Notably, recent advancements in the transcriptomic analysis of *K. fedtschenkoi* under various stress and growth conditions have provided new insights into the regulation of CAM photosynthesis in response to environmental stimuli, including light density and quality, drought, and high- and low-temperature stress [17,18,19]. Furthermore, a previous analysis of time-course transcriptomic data collected over one 12 h light/12 h dark (LD) cycle revealed both conservation and diversification of rhythmic regulatory mechanisms between the CAM model plant *K. fedtschenkoi* and the C_3_ model plant *Arabidopsis thaliana*. Functional conservation at the transcriptomic output level between the two species is sparse, and as expected, genes involved in carbon metabolism, circadian-regulated signaling pathways, and stomatal regulation exhibit extensive rhythmic rewiring in CAM species, consistent with the evolution of nocturnal CO_2_ fixation and inverted diel stomatal behavior [5,6,15].

However, the analysis of a single 24 h light/dark cycle is not sufficient to distinguish genes whose rhythmic expression is strictly driven by external light/dark cues from those that are maintained by the endogenous circadian rhythm under constant light conditions. To investigate the regulatory mechanisms driven by external light/dark cues and the internal circadian clock in *K. fedtschenkoi*, two sets of 48 h time-course transcriptomic data were generated under LD and LL conditions. Using these time-course data, genes rhythmically expressed under LD or LL were identified in *K. fedtschenkoi*. Furthermore, we analyzed the distribution of rhythmic genes associated with the CAM pathway, the circadian clock, and stomatal movement across the three light categories (LD, LL, and LD-LL). In addition, we constructed a co-expression network analysis of rhythmically expressed genes using 96 h time-course data (i.e., the 48 h LD time-course data combined with the 48 h LL time-course data) and characterized the co-expression modules enriched with genes relevant to the CAM pathway, stomatal movement, and the circadian clock.

We hypothesized that CAM transcriptional rhythmicity is largely governed by environmental light/dark cues, but a conserved circadian-controlled gene network provides essential temporal structure for CAM metabolism. Dissecting the interaction between these regulatory layers will reveal the fundamental mechanisms underlying the temporal optimization of CAM function. Our findings provide novel insights into the molecular mechanisms underlying rhythmic gene regulation, highlighting the distinct and overlapping roles of light/dark cues and circadian signaling in orchestrating temporal gene expression in *K. fedtschenkoi*.

## 2. Results

### 2.1. Rhythmical Expression Under LD and LL Conditions

**Figure 1:**
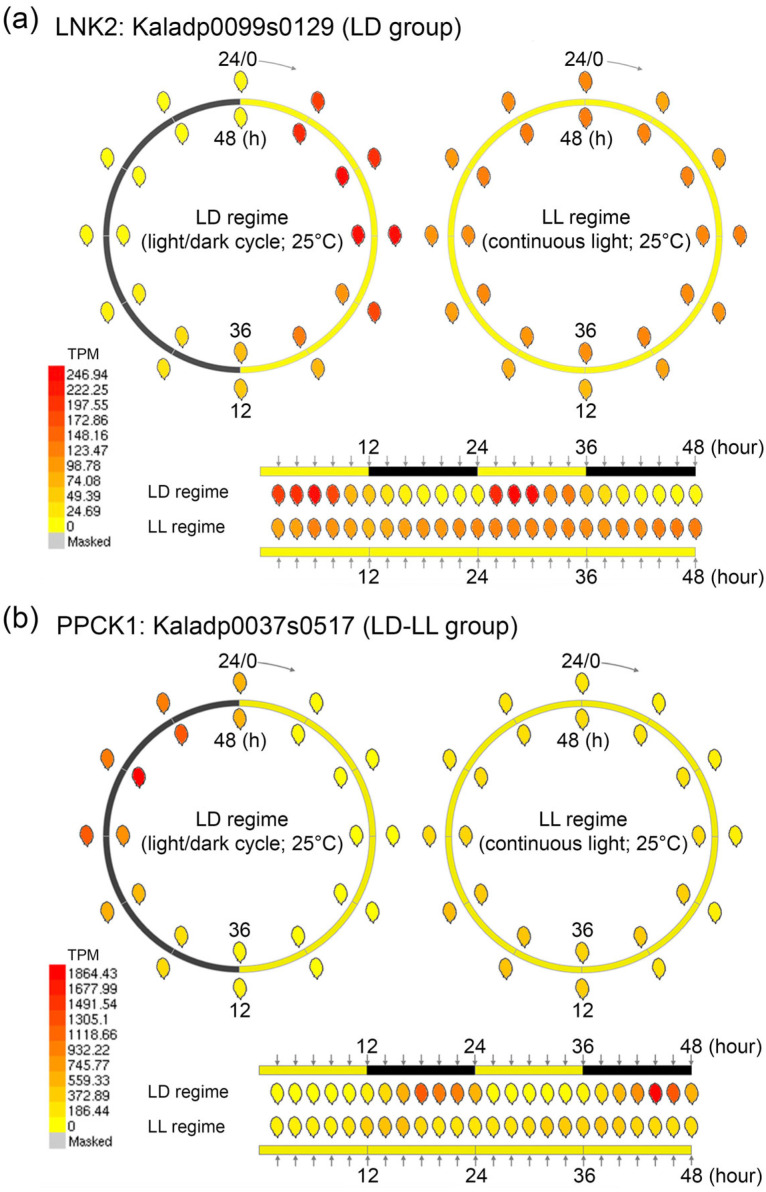
Overview of the eFP browser visualization for 48 h time-course transcriptomic datasets in *Kalanchoe fedtschenkoi* under light/dark (LD) and continuous light (LL) conditions. (**a**) Expression profile of LNK2 (Kaladp0099s0129) over a 48 h time course under LD and LL conditions. (**b**) Expression profile of PPCK1 (Kaladp0037s0517) over a 48 h time course under LD and LL conditions. LNK2, night light-inducible and clock-regulated gene 2; PPCK1, phosphoenolpyruvate carboxylase kinase 1. Yellow and black bars indicate daytime (12 h) and nighttime (12 h), respectively.

To explore gene expression dynamics with high temporal resolution, we developed an enhanced eFP (electronic Fluorescent Pictograph) browser integrating time-course transcriptomic datasets from *K. fedtschenkoi* (Figure 1). The browser features dynamic visualization tools, including synchronized time-lapse heatmaps and oscillatory trend graphs, enabling the systematic analysis of transcriptional responses under both 12 h/12 h light/dark (LD) cycles and continuous light (LL). This platform offers a robust framework for elucidating the molecular regulation and the circadian regulation in CAM species.

**Figure 2:**
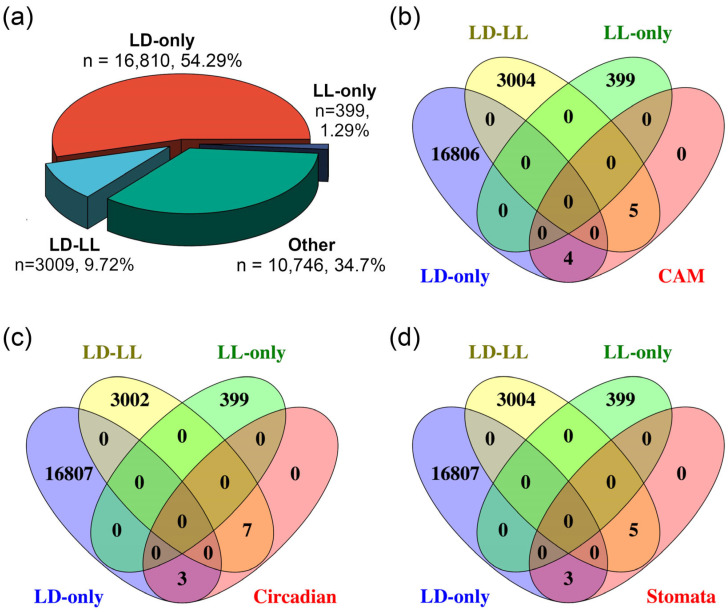
Rhythmic expression analysis for all transcripts and genes involved in CAM pathways, circadian clock signaling, and stomatal movement in *Kalanchoe fedtschenkoi*. (**a**) A pie chart diagram displaying percentage of all transcripts among various groups. (**b**) Venn diagram analysis for category identification of CAM-core genes. (**c**) Venn diagram analysis for category identification of circadian clock-related genes. (**d**) Venn diagram analysis for category identification of stomatal movement genes. LD-only: Rhythmic expression group containing genes showing rhythmic expression exclusively under the LD condition. LD-LL: Rhythmic expression group containing genes maintaining rhythmic expression under both LD and LL conditions. LL-only: Rhythmic expression group containing genes showing rhythmic expression exclusively under the LL condition.

Furthermore, we identified rhythmically expressed genes under LD or LL conditions using the JTK-CYCLE (JTK) algorithm implemented in the web-based circadian analysis tool Nitecap [20]. As illustrated in Figure 2a, among the 30,964 genes in *K. fedtschenkoi*, 20,218 genes exhibit 24 h oscillatory patterns, which can be divided into three groups: (1) the LD-only group (16,810 genes, 54.29% of all annotated protein-encoding genes in the genome), showing rhythmic expression exclusively under the LD condition; (2) the LL-only group (399 genes, 1.29%), with rhythmic expression only under the LL condition; and (3) the LD-LL group (3009 genes, 9.72%), maintaining rhythmicity under both conditions. These results suggest that the majority of genes were rhythmically regulated under LD, highlighting the critical role of light/dark cycles in maintaining proper rhythmic gene expression.

### 2.2. Rhythmic Gene Expression Patterns of CAM Pathway Genes

**Figure 3:**
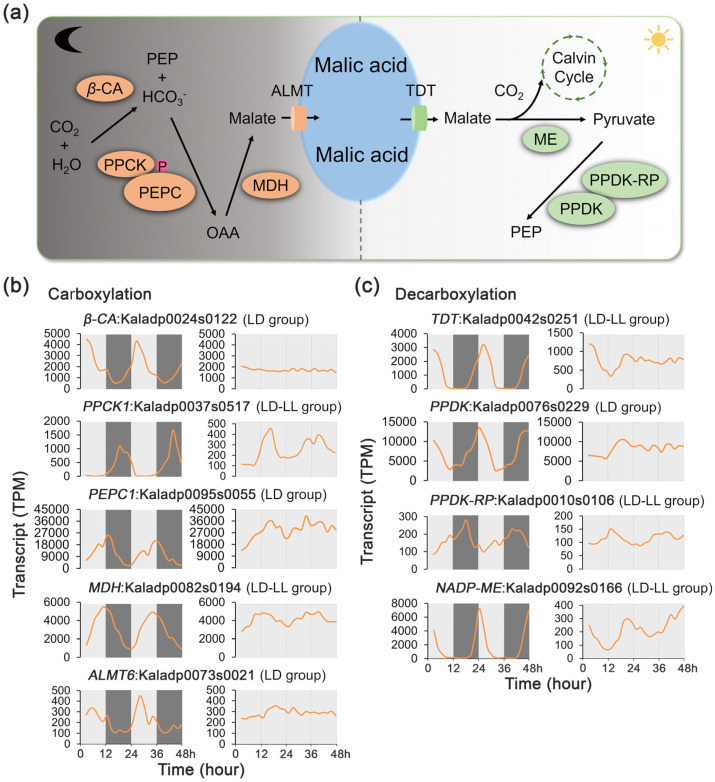
Transcript profiles of CAM photosynthetic regulatory genes under 48 h time-series light/dark (LD) and constant light (LL) conditions in *Kalanchoe fedtschenkoi*. (**a**) CAM photosynthetic pathway. (**b**) Expression patterns of genes involved in the CAM carboxylation process under LD and LL conditions. (**c**) Expression patterns of genes involved in CAM decarboxylation under LD and LL conditions. β-CA, β-carbonic anhydrase; PPCK1, phosphoenolpyruvate carboxylase kinase 1; PEPC, phosphoenolpyruvate carboxylase; MDH, NAD(P)-malate dehydrogenase; ALMT6, aluminum-activated malate transporter 6; TDT, tonoplast dicarboxylate transporter; NADP-ME, NADP-malic enzyme; PPDK, pyruvate phosphate dikinase; PPDK-RP, pyruvate orthophosphate dikinase-regulatory protein. LD-only: Rhythmic expression group containing genes showing rhythmic expression exclusively under the LD condition. LD-LL: Rhythmic expression group containing genes maintaining rhythmic expression under both LD and LL conditions. Orange lines represent transcript abundance. White and black bars indicate daytime (12 h) and nighttime (12 h), respectively.

The CAM pathway comprises two key biochemical processes: carboxylation followed by decarboxylation. The carboxylation process involves five key enzymes, including *β*-carbonic anhydrase (*β*-CA), PEPC, PEPC kinase (PPCK), NAD(P)-malate dehydrogenase (MDH), and the aluminum-activated malate transporter (ALMT) (Figure 3a; Supplementary Figure S1). The decarboxylation process of the pathway involves four key enzymes, including tonoplast dicarboxylate transporter (TDT), malic enzyme (ME), pyruvate phosphate dikinase (PPDK), and PPDK-regulatory protein (PPDK-RP) (Figure 3a). Among these nine known CAM pathway genes, four genes exhibited rhythmic expression exclusively under the LD condition, while five maintained rhythmicity under both LD and LL conditions. Notably, none of these CAM genes displayed rhythmic patterns solely under LL, underscoring the limited ability of the circadian clock to sustain CAM rhythmicity without external light/dark cues (Figure 2b, Supplementary Table S1).

In the carboxylation process, the transcripts of *β-CA* (Kaladp0024s0122), *ALMT6* (Kaladp0073s0021), and *PEPC1* (Kaladp0095s0055) exhibited a rhythmic pattern under LD but not LL (Figure 3b), indicating that the dynamics of these transcripts are controlled by external light/dark cues. On the other hand, the transcripts of *PPCK1* (Kaladp0037s0517) and *MDH* (Kaladp0082s0194) showed a rhythmic pattern under both LD and LL conditions, suggesting that the inherent circadian clock plays an important role in the regulation of these two transcripts.

In the decarboxylation process, the transcripts of two *PPDK* genes (Kaladp0039s0092) displayed a rhythmic pattern under LD but not LL (Figure 3c), indicating that the dynamics of these transcripts are controlled by external light/dark cues. In contrast, the transcripts of three other decarboxylation enzymes, *TDT* (Kaladp0042s0251), *PPDK-RP* (Kaladp0010s0106), and *NADP-ME* (Kaladp0092s0166), exhibited rhythmic patterns under both LD and LL conditions, although their peak abundances were significantly reduced after exposure to LL (Figure 3c), suggesting that the circadian clock plays an important role in the regulation of these three transcripts.

### 2.3. Rhythmic Gene Expression Patterns of Circadian Clock Genes

Plant circadian rhythms represent a complex array of regulatory mechanisms that underlie various developmental and physiological processes, as well as responses to environmental changes, such as light/dark growth cycles and temperature fluctuations [21,22,23]. In *Arabidopsis thaliana*, a number of regulatory components involved in the circadian clock pathways have been well documented, including signal sensing, central core oscillators, and signaling outputs [24].

**Figure 4:**
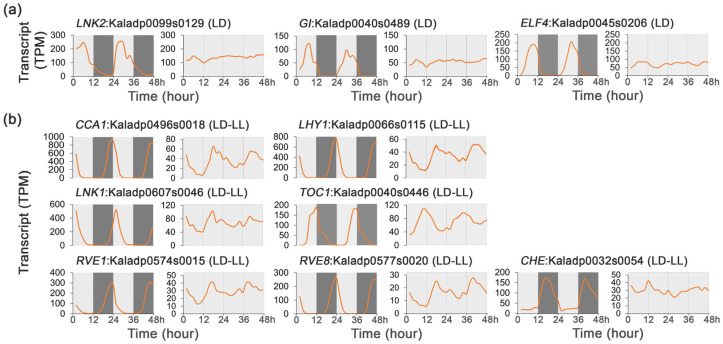
Transcript profiles of circadian clock core genes under 48 h time-series light/dark (LD) and constant light (LL) conditions in *Kalanchoe fedtschenkoi.* (**a**) Expression patterns of genes involved in circadian clock signaling under LD conditions. (**b**) Expression patterns of genes involved in circadian clock signaling under LD and LL conditions. CCA1, circadian clock associated 1; LHY1, late elongated hypocotyl 1; CHE, CCA1 hiking expedition; LNK1/2, night light-inducible and clock-regulated gene 1/2; RVE1/8, reveille 1/8; GI, gigantea protein; ELF4, early flowering 4; TOC1, timing of CAB expression 1. LD-only: Rhythmic expression group containing genes showing rhythmic expression exclusively under the LD condition. LD-LL: Rhythmic expression group containing genes maintaining rhythmic expression under both LD and LL conditions. Orange lines represent transcript abundance. White and black bars indicate daytime (12 h) and nighttime (12 h), respectively.

Based on the protein sequence similarity and gene expression patterns, a total of 10 circadian clock genes were identified in *K. fedtschenkoi*. Among these clock genes, three exhibited rhythmic expression exclusively under LD, and seven showed rhythmicity under both LD and LL (Figure 2c, Supplementary Table S1). Specifically, the transcripts of *LNK2* (Kaladp0099s0129), *GI* (Kaladp0040s0489), and *ELF4* (Kaladp0045s0206) exhibited rhythmic expression under LD but not LL (Figure 4a), indicating that the temporal dynamics of these genes are controlled by external light/dark cues.

The transcripts of *CCA1* (Kaladp0496s0018), *LHY1* (Kaladp0066s0115), *LNK1* (Kaladp0607s0046), *RVE1* (Kaladp0574s0015), *RVE8* (Kaladp0577s0020), *CHE* (Kaladp0032s0054), and *TOC1* (Kaladp0040s0446) exhibited rhythmic expression under both LD and LL (Figure 4b), suggesting that these gene are involved in the circadian system. Collectively, these results highlight the dual regulatory mechanisms regulating the expression of clock genes, wherein the endogenous circadian oscillator establishes a foundational rhythmicity, while external light/dark cues serve as the primary synchronizing signals. These environmental cues not only enhance the amplitude of rhythmic gene expression but also reprogram the transcriptional profiles to align with daily environmental changes.

### 2.4. Rhythmic Expression Patterns of Genes Involved in Stomatal Movement

Stomatal movement plays a critical role in regulating photosynthetic carbon assimilation, cellular respiration, and water-use efficiency in plants by mediating the exchange of carbon dioxide, oxygen, and water vapor between the plant and the atmosphere [25,26]. Notably, CAM species exhibit an inverted pattern of stomatal opening and closure compared to C_3_ and C_4_ plants, suggesting the presence of specialized regulatory signaling mechanisms governing stomatal movement [3].

To investigate the relationship between circadian rhythm and stomatal movement regulation, a Venn diagram analysis was performed to identify the overlap between rhythmic genes and genes involved in stomatal movement. Among the eight genes analyzed, three exhibited rhythmic expression under LD conditions, while another five retained rhythmicity under both LD and LL conditions (Figure 2d, Supplementary Table S1).

**Figure 5:**
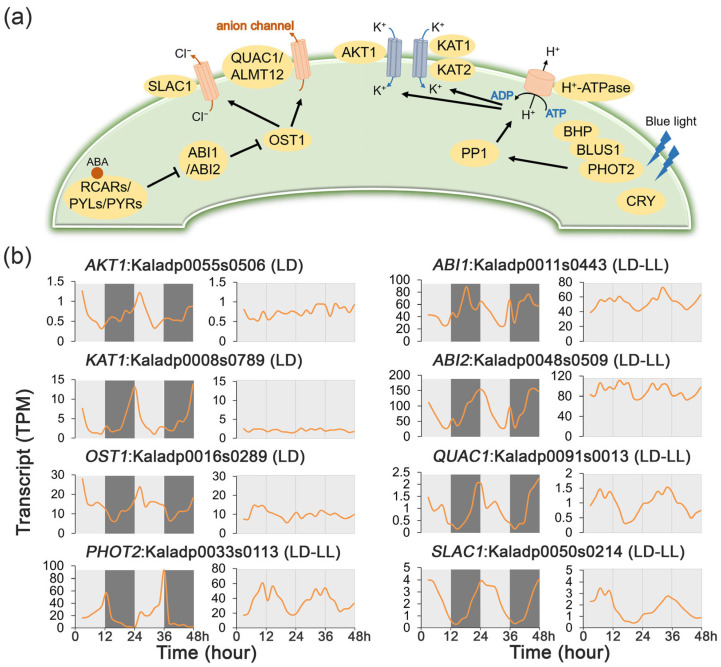
Transcript profiles of stomatal conductance regulatory genes under 48 h time-series light/dark (LD) and constant light (LL) conditions in *Kalanchoe fedtschenkoi.* (**a**) Plant stomatal movement regulatory pathway. (**b**) Expression patterns of genes involved in regulation of stomatal movement under LD and LL conditions. AKT1, potassium transporter 1; KAT1/2, potassium channel in Arabidopsis thaliana 1/2; OST1, open stomata 1; PHOT2, phototropin 2; ABI1/2, ABA insensitive 1/2; QUAC1, quick anion channel 1; BLUS1, blue light signaling 1; SLAC1, slow anion channel-associated 1. LD-only: Rhythmic expression group containing genes showing rhythmic expression exclusively under the LD condition. LD-LL: Rhythmic expression group containing genes maintaining rhythmic expression under both LD and LL conditions. Orange lines represent transcript abundance. White and black bars indicate daytime (12 h) and nighttime (12 h), respectively.

The expression dynamics of key stomatal regulatory genes were analyzed under LD and LL conditions to assess the transcriptional regulation (Figure 5b, Supplementary Figure S2). Circadian rhythmicity was observed for five genes, *ABI1* (Kaladp0011s0443), *ABI2* (Kaladp0048s0509), *QUICK ANION CHANNEL 1* (*QUAC1*, Kaladp0091s0013), *PHOTOTROPIN 2* (*PHOT2*, Kaladp0033s0113), and *SLOW ANION CHANNEL-ASSOCIATED 1* (*SLAC1*, Kaladp0050s0214), which maintained oscillations in both LD and LL. In contrast, the rhythmic expression of *AKT1* (Kaladp0055s0506), *KAT1* (Kaladp0008s0789), and *OST1* (Kaladp0016s0289) was conditional and dampened under LL, indicating their regulation is light-dependent rather than clock-driven. The constitutive circadian regulation of a core suite of stomatal genes likely underpins the inverted opening and closing pattern of stomatal conductance that defines CAM photosynthesis.

### 2.5. Rhythmic Expression Patterns of Genes Involved in Post-Transcriptional and Post-Translational Modifications

Post-translational modifications (PTMs) in plants are essential regulatory mechanisms that modulate protein activity, stability, localization, and interactions through rapid and often reversible chemical changes. In CAM species, PTM-mediated signaling has been shown to regulate gene expression and enzyme activity in a time-of-day-dependent manner, contributing to the temporal coordination of carbon metabolism with stomatal movement [8,9].

**Figure 6:**
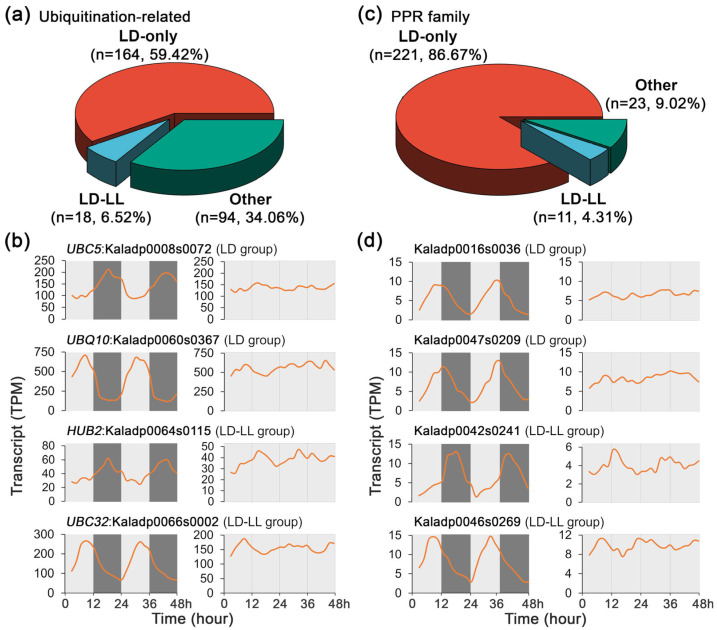
Transcript profiles of ubiquitination-related genes and PPR family genes under 48 h time-series light/dark (LD) and constant light (LL) conditions in *Kalanchoe fedtschenkoi.* (**a**) A pie chart diagram displaying percentage of ubiquitination-related genes among various groups. (**b**) Expression patterns of ubiquitination-related genes under LD and LL conditions. (**c**) A pie chart diagram displaying percentage of PPR-encoding genes among various groups. (**d**) Expression patterns of PPR-encoding genes under LD and LL conditions. LD-only: Rhythmic expression group containing genes showing rhythmic expression exclusively under the LD condition. LD-LL: Rhythmic expression group containing genes maintaining rhythmic expression under both LD and LL conditions. UBC5/32, ubiquitin-conjugating enzyme 5/32; UBQ10, ubiquitin 10; HUB2, histone mono-ubiquitination 2. Orange lines represent transcript abundance. White and black bars indicate daytime (12 h) and nighttime (12 h), respectively.

To investigate the roles of PTMs in the regulation of CAM physiology, the transcript profiles of ubiquitination-related genes were analyzed under LD and LL conditions, which play central roles in regulating diverse biological processes, including hormone signaling, stress responses, development, and immune protection. Of the 278 ubiquitination-related genes, 164 (59.42%) exhibited rhythmic expression exclusively under LD conditions, whereas none displayed rhythmic expression exclusively under LL (Figure 6a). Notably, 18 (6.52%) maintained rhythmicity under both LD and LL conditions, although a reduction in transcript peak amplitudes under LL compared to LD was observed for the ubiquitination-related genes (Figure 6b).

In addition, the transcripts of the pentatricopeptide repeat (PPR) protein encoding genes, which are characterized by tandem repeats of a conserved 35-amino acid motif, were investigated under LD and LL conditions. Within the PPR gene family detected in *K. fedtschenkoi*, 221 out of 255 genes (86.67%) exhibited rhythmic expression exclusively under LD, with only 11 genes retaining rhythmicity under both LD and LL conditions. No PPR gene showed rhythmic expression under LL alone (Figure 6c). A reduction in transcript peak amplitudes was also observed under LL compared to LD (Figure 6d). Our findings further reinforce the distinct regulatory mechanisms governing gene expression in CAM species, where diurnal light environmental signals play a dominant role in modulating gene expression, while internal circadian regulation maintains a subset of rhythmic patterns under constant light conditions.

### 2.6. Co-Expression Network of Rhythmic Expression Genes

To investigate co-expression regulation in *K. fedtschenkoi*, a weighted gene co-expression network analysis (WGCNA) [27] was performed using all the identified rhythmically expressed genes in the LD-only, LL-only, and LD-LL groups. In total, we identified 42 distinct co-expression modules, each represented by a unique color (Supplementary Figures S3 and S4). The heatmap visualizations revealed independent expression patterns and correlations among these modules, highlighting the diversity of transcriptional regulation across the expression network (Supplementary Figure S5).

**Figure 7:**
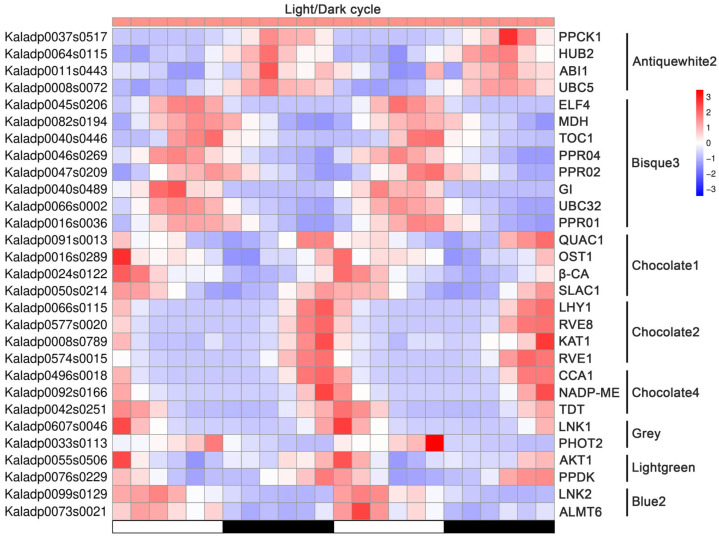
Expression pattern of genes involved in CAM regulation, circadian signaling, stomatal movement, ubiquitination, and the PPR family under light/dark cycle in *Kalanchoe fedtschenkoi*. Z-score was used for normalization of gene transcript profiles and for generation of the heatmap. β-CA, β-carbonic anhydrase; PPCK1, phosphoenolpyruvate carboxylase kinase 1; MDH, NAD(P)-malate dehydrogenase; ALMT6, aluminum-activated malate transporter 6; TDT, tonoplast dicarboxylate transporter; NADP-ME, NADP-malic enzyme; PPDK, pyruvate phosphate dikinase; CCA1, circadian clock associated 1; LHY1, late elongated hypocotyl 1; LNK1/2, night light-inducible and clock-regulated 1/2; RVE1/8, reveille 1/8; GI, gigantea protein; ELF4, early flowering 4; TOC1, timing of CAB expression 1; AKT1, potassium transporter 1; KAT1, potassium channel in Arabidopsis thaliana 1; OST1, open stomata 1; PHOT2, phototropin 2; ABI1, ABA insensitive 1; QUAC1, quick anion channel 1; SLAC1, slow anion channel-associated 1; UBC5/32, ubiquitin-conjugating enzyme 5/32; HUB2, histone mono-ubiquitination 2. White and black bars indicate daytime (12 h) and nighttime (12 h), respectively.

Furthermore, we examined the distribution of genes involved in the CAM metabolic pathway, circadian signaling, stomatal movement, ubiquitination, and the PPR family across the co-expression modules. As illustrated in Figure 7, the genes from the diverse signaling pathways were co-expressed within the same modules, indicating potential regulatory interactions. For instance, in the module ‘Antiquewhite2’, *PPCK1* and *ABI1* were co-expressed with the ubiquitination-related genes *HUB2* and *UBC5*. In ‘Bisque3’, the circadian clock genes *ELF4*, *TOC1*, and *GI* shared similar expression patterns with the PPR family genes (*PPR01*, *PPR02*, *PPR04*) and the CAM-related gene *MDH*. These results suggest an integrated regulatory network linking CAM metabolism, circadian rhythms, stomatal regulation, and post-translational modifications in CAM species. Notably, distinct modules revealed strong associations between circadian and functional CAM or stomatal genes: in module ‘Chocolate1’, the circadian regulators *QUAC1*, *OST1*, and *SLAC1* were co-expressed with *β-CA*; in module ‘Chocolate2’, *LHY1*, *RVE1*, and *RVE8* clustered with the stomatal movement gene *KAT1*; and in module ‘Chocolate4’, *CCA1* was co-expressed with the CAM genes *TDT* and *NADP-ME*. Collectively, these findings suggest that circadian signaling acts as a central hub integrating CAM metabolism, stomatal regulation, and post-translational modifications in CAM species, facilitating the temporal coordination of the physiological processes critical for adaptation to arid environments.

Additionally, co-expression subnetworks for CAM metabolic genes, circadian clock genes, and stomatal movement genes were extracted using a stringent threshold for the Pearson correlation coefficient (|PCC| > 0.85, *p* ≤ 0.05) to identify robust co-expression relationships (Supplementary Table S2). Notably, *ALMT6* (Kaladp0073s0021) exhibited co-expression within the ‘blue2’ module, including with two transcription factors, *PRR3* (Kaladp0101s0041) and *PRR7* (Kaladp0099s0080), as well as *HEAT SHOCK PROTEINS 60* (*HSP60*, Kaladp0011s0447), *HSP89.1* (Kaladp0033s0317), *MITOGEN-ACTIVATED PROTEIN KINASE 15* (*MPK15*, Kaladp0095s0820), *MPK16* (Kaladp0055s0555), and *CHLORIDE CHANNEL C* (*CLC-c*, Kaladp0079s0096) (Supplementary Figure S6b). Regarding the circadian clock-related subnetworks (Supplementary Figure S6c), *CCA1* and *LHY1* were tightly co-expressed and displayed strong associations with several genes, including *POTASSIUM TRANSPORT 2/3* (*AKT2/3*, Kaladp0062s0205), *RVE1* (Kaladp0574s0015), *RVE6/8* (Kaladp0577s0020), and *CATALASE 2* (*CAT2*, Kaladp0052s0025). In addition, *LHY1* was co-expressed with *RVE7* (Kaladp0262s0013) and a PP2C encoding gene (Kaladp0515s0184) (Supplementary Figure S6c). We also characterized the co-expression network of *OST2* (Kaladp0098s0188), which showed strong co-expression with *INDUCER OF CBF EXPRESSION 1* (*ICE1*, Kaladp0016s0053) and *SALT OVERLY SENSITIVE 1* (*SOS1*, Kaladp0095s0179) (Supplementary Figure S6d).

### 2.7. Hub Genes Relevant to CAM Regulation

Through the WGCNA analysis, hub genes, characterized by their high interconnectivity within co-expression modules, were identified, representing central regulatory elements in various biological processes and molecular networks. As shown in Table 1, the analysis revealed two key metabolic enzymes, PEPC1 (Kaladp0095s0055) in the carboxylation process and PPDK (Kaladp0076s0229) in the decarboxylation process, as hub genes in the ‘darkgrey’ and ‘lightgreen’ modules, respectively. These findings strongly support their pivotal roles in orchestrating CAM photosynthesis, consistent with their demonstrated functions in carbon fixation and metabolic flux.

Notably, the co-expression network analysis uncovered two circadian clock regulators, RVE1 (Kaladp0574s0015) and RVE8 (Kaladp0577s0020), as hub genes within the ‘chocolate2’ module, suggesting that these transcription factors act as central coordinators of the circadian regulatory network in CAM species, potentially mediating the temporal control of CAM-related gene expression.

A particularly intriguing discovery was the prominent involvement of PTM machinery in the regulation of CAM. Multiple ubiquitination-related genes, including E3 ubiquitin ligases (HUB2, Kaladp0064s0115) and ubiquitin-conjugating enzymes (UBC5, Kaladp0008s0072 and UBC32, Kaladp0066s0002) emerged as hub genes within the ‘antiquewhite2’ and ‘bisque3’ modules. In addition, PPR-encoding genes were also identified as network hubs in the ‘lightpink3’ and ‘bisque3’ modules (Table 1). These findings collectively highlight the crucial role of protein turnover and post-translational regulation in modulating CAM pathway activity, suggesting sophisticated processes of regulatory control beyond transcriptional regulation. The prevalence of these modification systems as network hubs implies their involvement in regulating CAM-related processes in response to environmental and endogenous signals.

## 3. Discussion

Plants performing CAM photosynthesis have evolved a sophisticated biochemical and regulatory system that optimizes carbon gain while minimizing water loss, making them exceptionally well-adapted to arid and semi-arid environments. CAM hinges on a strict temporal separation of physiological processes, primarily nocturnal stomatal opening and CO_2_ uptake, followed by daytime malate decarboxylation and CO_2_ refixation behind closed stomata [1,2,15,28]. In this study, by generating high-resolution 48 h time-course transcriptomic data from the model CAM species *Kalanchoe fedtschenkoi* under both light/dark (LD) cycles and continuous light (LL), we provide a comprehensive view of the transcriptional architecture governing the dynamic CAM physiology. These findings reveal that the daily light/dark cycle is the dominant driver of rhythmic gene expression in *K. fedtschenkoi* and that the endogenous circadian clock acts as a master coordinator, integrating environmental cues with metabolic and developmental programming through multiple, previously unrecognized regulatory layers, including post-transcriptional and post-translational controls.

### 3.1. The Primacy of Diel Cues in Orchestrating the CAM Transcriptome

Rhythmic gene expression in plants is regulated by both the endogenous circadian clock and external environmental cues, aligning biological processes with the 24 h cycle for optimizing resource use [29,30]. A fundamental question in circadian biology is the extent to which rhythmic physiological processes are driven by the endogenous, self-sustaining oscillator versus being a direct response to external environmental cycles [31]. Our results provide a clear and quantitative answer for a CAM plant: the *K. fedtschenkoi* transcriptome is overwhelmingly governed by diel cues. Of the nearly 20,000 genes exhibiting rhythmic expression, the vast majority, 16,810 genes (54.3% of all protein-encoding genes in the genome), were rhythmic exclusively under LD conditions, losing their rhythmicity in constant light (Figure 2a). This is consistent with observations in the C_3_ model plant *Arabidopsis*, where more transcripts oscillate under LD cycles than in constant light [32]. This pattern of diel dominance extends across all key functional pathways underpinning CAM physiology. Core CAM pathway genes, circadian clock components, and stomatal movement regulators were all predominantly found in the LD-only and LD-LL groups, with very few being rhythmic under LL alone (Figure 2, Figure 3, Figure 4 and Figure 5). Notably, for the genes in the LD-LL group, the transcript peak amplitudes were consistently and markedly attenuated under LL conditions. These observations demonstrate that although the endogenous clock establishes a foundational rhythmic signal, the daily light–dark transition is crucial for amplifying gene expression to the levels needed to support the massive metabolic fluxes characteristic of CAM, thereby reframing the CAM regulatory system as one that depends on strong, daily reinforcement from environmental cues.

Furthermore, light/dark cycling promotes coherent rhythmic regulation of CAM-associated signaling pathways, whereas continuous light disrupts this coordination, leading to phase shifts and transcript attenuation in gene expression and extensive network rewiring (Figure 3, Figure 4, Figure 5 and Figure 6). This observation is consistent with recent transcriptomic analyses in *Arabidopsis thaliana*, which demonstrate that light regimes strongly shape circadian gene co-expression networks and rhythmic transcriptional outputs, even when the core oscillator remains intact [32]. High-resolution time-series transcriptomic studies further indicate that diel light cycles reinforce temporal alignment across metabolic and signaling pathways through intrinsic circadian control [33]. Moreover, dynamic light environments enable plants to balance photoprotection, metabolic efficiency, and temporal coordination, highlighting rhythmic regulation as a key determinant of long-term performance and stress resilience in crops [34,35]. Collectively, these studies support a model in which alternating light regimes enhance CAM fitness by stabilizing coordinated gene expression programs that optimize nocturnal carbon uptake, daytime decarboxylation, and stomatal control, thereby enhancing crop adaption and tolerance to abiotic stress, an insight with important implications for CAM-C_3_ engineering strategies.

### 3.2. The Circadian Clock as a Master Coordinator of Metabolism and Stomatal Gating in CAM

Our network analysis positions the circadian clock as a central hub coordinating the temporal regulation of the entire CAM system. Core clock components (*CCA1*, *LHY1*, *RVE1*, *RVE8*, *TOC1*) were consistently co-expressed with key CAM metabolic genes (*PPCK1*, *MDH*, *TDT*, *NADP-ME*) and stomatal regulators (*KAT1*, *SLAC1*, *OST1*) across multiple modules (Figure 7), highlighting a tightly integrated regulatory architecture. Consistent with the concept of ‘circadian gating’, these results indicate that the clock not only tracks time but also modulates plant responsiveness to light signals [7].

Light acts as a strong transcriptional regulator independent of the circadian clock, while the clock restricts these responses to the appropriate phases of the day [36,37,38]. Our data support a model in which the circadian oscillator establishes a ‘permissive window’, particularly near dusk, during which the light-responsive transcriptional programs required for nocturnal CO_2_ uptake are primed. The subsequent transition to darkness then triggers the high-amplitude expression of CAM-associated genes. This gating mechanism explains why a large fraction of the transcriptome exhibits rhythmicity only under LD conditions: their expression is driven by light-to-dark transitions but is permitted only at specific circadian phases. Consistent with this model, continuous light dampened both the prevalence and the amplitude of rhythmic CAM-related transcripts. Although some circadian oscillations persisted under LL, they frequently displayed altered phases or reduced amplitudes, indicating that diel light cues actively shape clock-regulated transcriptional outputs.

The importance of light entrainment was further supported by the analysis of the MYB-family transcription factors, key components of the plant circadian oscillator [23]. Among the 334 detected MYB genes in *K. fedtschenkoi* (Supplementary Figure S7; Supplementary Table S3), 146 exhibited 24 h rhythmicity under LD, whereas only 5 were rhythmic exclusively under LL, and 49 oscillated under both conditions. Notably, the MYB transcript amplitudes were markedly reduced under LL. This significant contrast underscores the requirement of cyclic environmental cues for robust activation of clock-associated transcription factors and reinforces the central role of light entrainment in sustaining the CAM circadian network.

### 3.3. A Novel Tier of CAM Regulation: Multi-Level Post-Transcriptional and Post-Translational and Control

The most significant and novel finding of this study is that CAM regulation extends well beyond transcription to include coordinated post-translational and post-transcriptional control. The co-expression network analysis identified multiple ubiquitination- and RNA-processing-related genes as central hubs within the CAM-associated modules, revealing additional regulatory layers essential for temporal precision. Notably, the E3 ubiquitin ligase HUB2 and ubiquitin-conjugating enzymes UBC5 and UBC32 emerged as prominent hubs (Table 1). The co-expression of HUB2 and UBC5 with the core CAM kinase PPCK1 and the ABA signaling component ABI1 in the antiquewhite2 module (Figure 7) suggests a dual regulatory mechanism. In C_4_ plants, the PPCK protein is rapidly degraded via the ubiquitin–proteasome pathway to enable swift metabolic transitions [39]. Our co-expression network analysis provides the first transcriptome-level evidence in a CAM species that PPCK1 is tightly associated with ubiquitination-related genes, implicating a two-tiered regulatory model in which circadian control of PPCK1 transcription is coupled with ubiquitin-mediated protein turnover, potentially enabling rapid inactivation of PPCK1 and preventing futile phosphorylation cycles.

More fundamentally, the identification of HUB2 implicates epigenetic regulation as an integral component of CAM control. Notably, HUB2 is a well-characterized E3 ligase involved in histone modification, catalyzing the mono-ubiquitination of histone H2B (H2Bub1), a chromatin modification associated with transcriptional activation and downstream H3K4 methylation [40,41]. This modification promotes downstream histone methylation and facilitates dynamic gene expression in response to developmental signals and environmental stimuli [40,42]. Its co-expression with *PPCK1* and *ABI1* supports a model in which the circadian clock modulates HUB2 activity to epigenetically prime CAM- and stomata-related loci, restricting transcriptional competence to appropriate diel phases. Such a mechanism is consistent with the circadian–epigenetic coupling described in C_3_ species, in which clock components modulate histone modification landscapes to coordinate metabolic processes and environmental responsiveness [43,44]. Our data extend this paradigm to CAM species, suggesting that epigenetic regulation contributes to the precise temporal alignment of nocturnal carbon fixation, daytime decarboxylation, and stomatal behavior that defines CAM physiology. Taken together, these findings reveal a completely new tier of CAM regulation, such that the circadian clock prepares the chromatin landscape in advance of diel transitions, facilitating robust and phase-specific transcriptional responses.

In parallel, pentatricopeptide repeat (PPR) proteins were identified as central hubs in the CAM regulatory networks. PPR proteins function as site-specific RNA-binding factors involved in RNA editing, splicing, stability, and translation, primarily within mitochondria and chloroplasts [45,46]. PPR hubs in modules such as *bisque3* were co-expressed with clock genes (*TOC1*, *GI*) and the CAM enzyme *MDH* (Figure 7; Table 1). Given the substantial organellar metabolic demands of CAM physiology [3], these findings suggest that the circadian clock coordinates nuclear and organellar gene expression via PPR-mediated RNA regulation. Collectively, these results uncover previously unappreciated regulatory tiers, epigenetic priming, and organellar RNA control, which are essential for the temporal orchestration of CAM metabolism.

### 3.4. An Updated Model for the Molecular Regulation of CAM Photosynthesis

**Figure 8:**
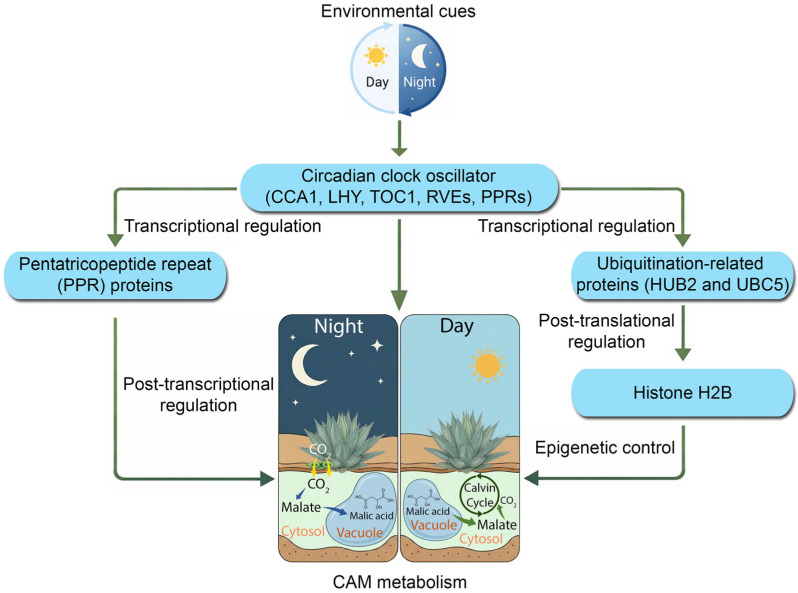
A hypothetic model of molecular regulation of CAM physiology to environmental light/dark cycles in *Kalanchoe fedtschenkoi*. Histone H2B, a core component of the nucleosome (the basic structural unit of chromatin in eukaryotic cells); CCA1, circadian clock associated 1; LHY, late elongated hypocotyl; HUB2, histone mono-ubiquitination 2; PPR, pentatricopeptide repeat; PRRs, pseudo-response regulators; RVEs, REVEILLE transcription factors; TOC1, timing of cab expression 1; UBC5, ubiquitin-conjugating enzyme 5.

Collectively, our findings facilitate an updated and more comprehensive model of CAM regulation (Figure 8). The previous model often depicted a simple feedback loop where the circadian clock directly regulates the transcription of CAM genes. For example, cis-regulatory motif enrichment analyses in pineapple revealed that many CAM pathway genes harbor promoter elements bound by core clock regulators such as CCA1 and LHY, suggesting circadian gating of carbon fixation and malate metabolism [47]. In the facultative CAM species *Sedum album*, the transition from C_3_ to CAM under drought stress involves the large-scale reprogramming of diel expression networks, including shifts in circadian gene phase and amplitude that coordinate carbohydrate and malate metabolism with stomatal conductance [48]. However, our data reveal a far more intricate, multi-layered regulatory architecture (Figure 8). In this revised model, strong diel inputs from the light/dark cycle act as the primary entrainment signal for the entire system. This environmental information is processed by a central circadian oscillator (comprising CCA1/LHY, RVEs, PRRs, etc.), which in turn acts as a master gating mechanism. The clock’s output is then channeled through at least four distinct regulatory layers: (1) direct transcriptional control of CAM, stomatal, and other regulatory genes; (2) epigenetic control, mediated by hubs such as HUB2, which modulate chromatin accessibility; (3) post-transcriptional organellar control, mediated by PPR protein hubs, which synchronize mitochondrial and chloroplast function with the demands of the nuclear-driven CAM cycle; and (4) post-translational control, mediated by ubiquitination hubs such as UBC5, which govern protein stability and turnover.

## 4. Materials and Methods

### 4.1. Plant Material and Sample Collection

Freshly excised young stem cuttings of *Kalanchoe fedtschenkoi* (ORNL diploid accession M2) were used for vegetative propagation and grown individually in pots. Plants were cultivated in a controlled-environment growth chamber (Percival Model AR-75L2) for four weeks to ensure uniform establishment (Supplementary Figure S8). Approximately 200 plants were initially grown under white light at a photosynthetic photon flux density (PPFD) of 250 μmol m^−2^ s^−1^ (measured at the plant height), with a 12 h light (25 °C)/12 h dark (18 °C) photoperiod and relative humidity maintained at approximately 50% [15,49].

For the experiment itself, two groups of plants were maintained at a constant 25 °C under two distinct lighting regimes. One group (under the LD regime) experienced a 12 h light (250 μmol m^−2^ s^−1^)/12 h dark cycle for 16 days. The second group (under the LL regime) was subjected to the same 12 h light (250 μmol m^−2^ s^−1^)/12 h dark cycle for 14 days, followed by transfer to continuous light for an additional four days at a reduced light intensity of 100 μmol m^−2^ s^−1^ to minimize photoinhibition while maintaining circadian free-running conditions. Mature leaf samples (leaf pairs 5–7 counting from the top) were collected from distinct plants every two hours over a 48 h period during the final two days of each experimental lighting condition. All samples were immediately frozen in liquid nitrogen and stored at −80 °C for subsequent RNA extraction. Three individual plants were sampled at each time point, representing three biological replicates per treatment.

### 4.2. Total RNA Extraction

Total RNAs were extracted from approximately 100 mg of pulverized cryogenically frozen leaf tissue following the protocols described by Hu et al. [18,19]. In particular, the ground tissue was first suspended in 850 μL of CTAB buffer with freshly added 1.0% *β*-mercaptoethanol and incubated in a 56 °C water bath for 5 min with continuous mixing on a shaker incubator. The sample was then thoroughly mixed with 600 μL chloroform/isoamyl alcohol (24:1) and centrifuged at maximum speed for 8 min at room temperature. The upper aqueous phase was carefully transferred to an RNA binding column supplied by the Sigma Spectrum™ Plant Total RNA Kit (Sigma, Cat. No. STRN250-1KT, St. Louis, MO, USA) and spun at full speed for 1 min. The filtrate was mixed with 500 μL of 100% EtOH and applied to a binding column. Subsequent RNA extraction steps were carried out according to the manufacturer’s instructions. An on-column DNase treatment was then performed to remove residual genomic DNA contamination. RNA quality was assessed using a NanoDrop 1000 spectrophotometer (Thermo Scientific, Wilmington, DE, USA). A total of 3 µg RNA per sample was sent to HudsonAlpha Institute for Biotechnology (Huntsville, AL, USA) for library preparation and transcriptomic sequencing.

### 4.3. Library Preparation and RNA Sequencing

Plate-based RNA sample preparation was performed on a PerkinElmer Sciclone NGS robotic liquid handling system using Illumina’s TruSeq Stranded mRNA HT sample preparation kit utilizing poly-A selection of mRNA following the protocol outlined by Illumina (San Diego, CA, USA) in their user guide: https://support.illumina.com/sequencing/sequencing_kits/truseq-stranded-mrna.html (accessed on 25 September 252025), and with the following conditions: total RNA starting material was 1 ug per sample and 8 cycles of PCR were used for library amplification. The prepared libraries were quantified using KAPA Biosystem’s next-generation sequencing library qPCR kit (Roche, Basel, Switzerland) and run on a Roche LightCycler 480 real-time PCR instrument. The quantified libraries were then multiplexed and the pool of libraries was prepared for sequencing on an Illumina HiSeq sequencing platform utilizing a TruSeq paired-end cluster kit, v4, and Illumina’s cBot instrument to generate a clustered flow cell for sequencing. Sequencing of the flow cell was performed on an Illumina HiSeq 2500 sequencer using HiSeq TruSeq SBS sequencing kits and v4, following a 2 × 150 indexed run recipe.

### 4.4. Data Analysis

Following the removal of low-quality reads, RNA-seq reads from each library were aligned to the *K. fedtschenkoi* reference genome using GSNAP (v2018-07-04) [15]. Raw gene counts were generated using the FeatureCounts function in Subread (v1.6.1) [50], with only uniquely mapped reads to a single genomic locus included in the analysis. Gene expression levels were quantified as transcripts per million (TPM) [51]. Rhythmic expression related genes in a 24 h cycle were identified using the JTK_CYCLE algorithm implemented in the web tool Nitecap [20], with a significance threshold *q*-value < 0.05. A weighted gene co-expression network analysis (WGCNA) was performed using log_2_-transformed transcripts per million (TPM) values from all samples via an all-in-one R (version 4.4)/Bioconductor BioNERO (Biological Network Reconstruction Omnibus) package (version 1.18.0) [27,52], with the parameters of net_type = ‘signed hybrid’, SFTpower = 9, and cor_method = ‘biweight’. The SFTpower was determined based on scale-free topology fitting using the SFT_fit function. An electronic Fluorescent Pictograph (eFP) browser visualization was generated through the Bio-Analytic Resource (BAR) using TPM-normalized values of the time-course RNA-seq datasets [53]. All software were run with default parameters.

## 5. Conclusions

In summary, this high-resolution temporal transcriptome analysis provides the first comprehensive insight into rhythmic gene regulation in a CAM plant species under both entrained day/night and free-running conditions. Our results demonstrate that environmental light/dark cues exert a dominant influence on the *K. fedtschenkoi* transcriptome, driving robust 24 h oscillations in thousands of genes, including those for CAM photosynthesis, stomatal control, circadian clock components, and regulators of transcription and protein modifications. In contrast, the endogenous circadian clock alone sustains rhythmic expression in only a limited subset of genes (often with diminished amplitude), underscoring the necessity of diel light/dark signals for the full expression of CAM’s temporal programs. Light-driven re-entrainment appears critical for reprogramming transcriptional rhythms and amplifying gene expression peaks, thereby fine-tuning CAM metabolic and physiological processes to daily cycles.

This integrated, multi-tiered system ensures the robust, high-amplitude, and temporally precise execution of CAM physiology, allowing plants such as *K. fedtschenkoi* to thrive in challenging environments. These findings substantially advance our understanding of how CAM plants integrate external and internal cues to regulate their metabolism over the day–night cycle. The co-expression network analysis, in particular, reveals previously unrecognized connections between CAM pathway genes and circadian regulators with post-translational machinery, suggesting new points of control in the CAM regulatory hierarchy and offering new routes to CAM engineering. Specifically, the molecular players and network hubs identified in this study serve as potential targets for crop improvement. By leveraging the clock and signaling components that enhance water-use efficiency in CAM species, researchers can explore strategies to introduce or bolster CAM-like water-saving traits in conventional C_3_ crops. Ultimately, the knowledge gained here lays a foundation for engineering improved water-use-efficient photosynthesis in non-CAM plants, addressing agricultural sustainability in water-limited environments.

## Figures and Tables

**Table 1:** Hub genes identified in co-expression modules relative to CAM photosynthesis.

Gene_ID	Name	Category	Module
Kaladp0095s0055	*PEPC1*	CAM	darkgrey
Kaladp0076s0229	*PPDK*	CAM	lightgreen
Kaladp0574s0015	*RVE1*	Circadian Clock	chocolate2
Kaladp0577s0020	*RVE8*	Circadian Clock	chocolate2
Kaladp0008s0072	*UBC5*	Ubiquitination	antiquewhite2
Kaladp0064s0115	*HUB2*	Ubiquitination	antiquewhite2
Kaladp0066s0002	*UBC32*	Ubiquitination	bisque3
Kaladp0016s0036	*PPR encoding gene 1*	PPR Family	bisque3
Kaladp0042s0241	*PPR encoding gene 3*	PPR Family	lightpink3
Kaladp0046s0269	*PPR encoding gene 4*	PPR Family	bisque3

## Data Availability

The data presented in this study are openly available in [NCBI] at [https://www.ncbi.nlm.nih.gov/sra/?term=Kalanchoe+fedtschenkoi+M2+Circadian+control (accessed on 21 December 2025)], reference number [SRX4085178–SRX4085321].

## Supplementary Materials

The following supporting information can be downloaded at: https://www.mdpi.com/article/10.3390/ijms27031342/s1.
