# Supplementary material for: Rhythmic Mechanisms Governing CAM Photosynthesis in Kalanchoe fedtschenkoi: High-Resolution Temporal Transcriptomics
Source: Int J Mol Sci. 2026 Jan 29;27(3):1342. doi: 10.3390/ijms27031342 (PMC12897691; doi:10.3390/ijms27031342)
Supplement: Supplementary file 1 [file ijms-27-01342-s001.zip › Supplementary Figures.pdf]

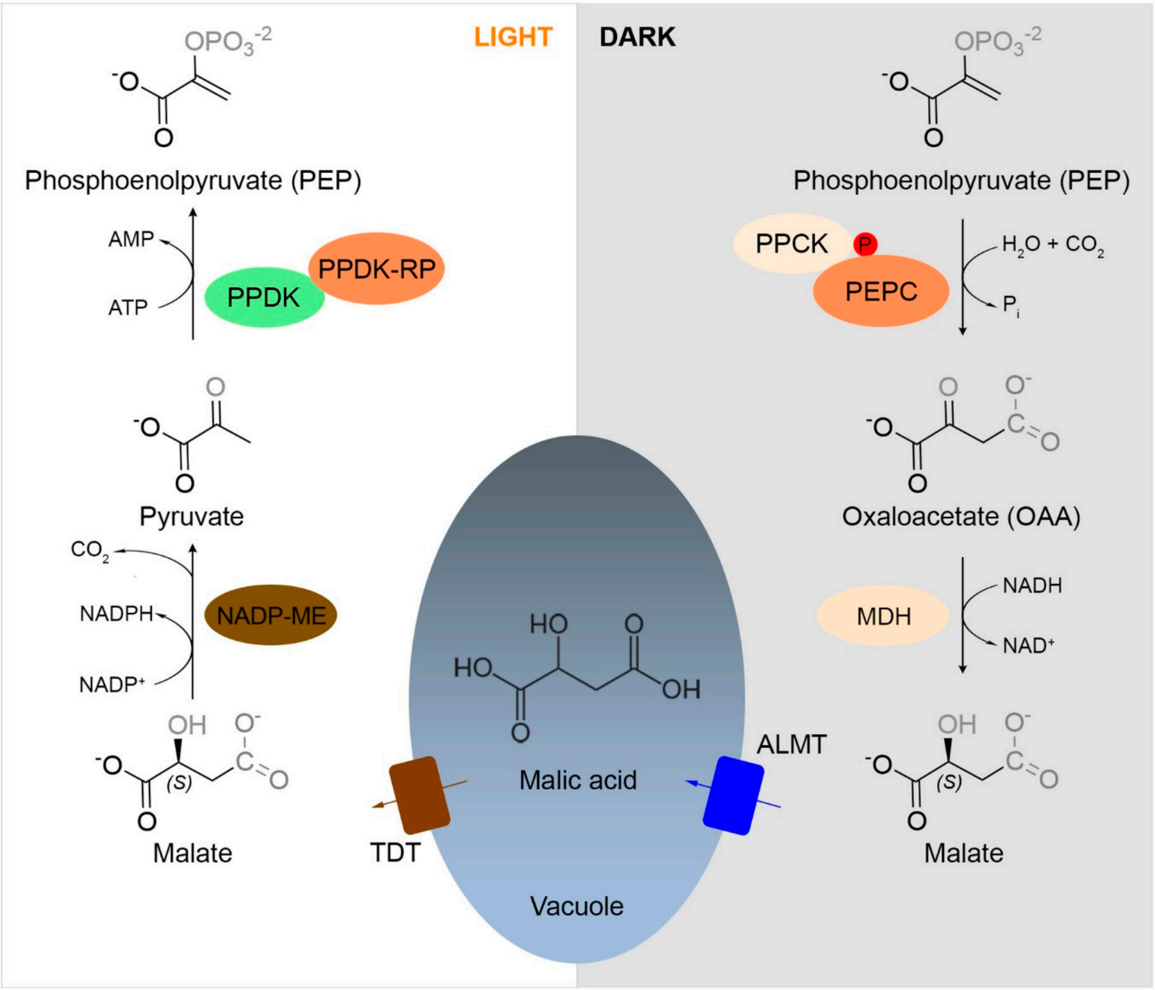

Supplementary Figure S1. Overview of the CAM metabolic pathway, with chemical structures shown for key compounds. Enzymes are labeled using the same color scheme as in Figure 7 for consistency.

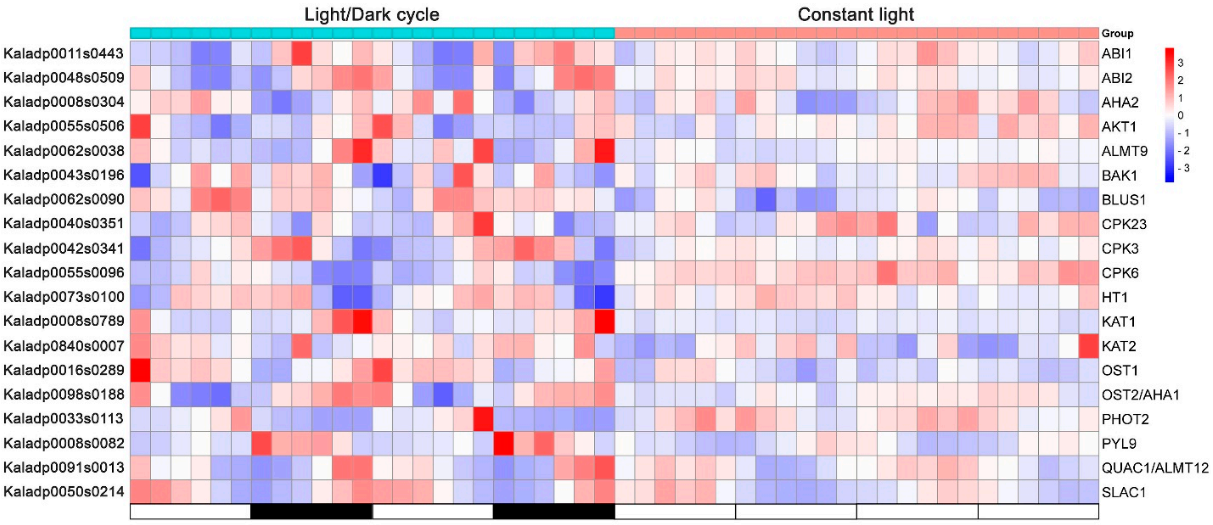

Supplementary Figure S2. Expression pattern of genes involved in stomatal movement under light/dark cycle in *Kalanchoe fedtschenkoi*. Z-score was used for normalization of gene transcript profiles and for generation of the heatmap. ABI1/2, abscisic acid insensitive 1/2; AHA1/2, Arabidopsis H^+-ATPase 1/2; AKT1, Arabidopsis K^+ transporter 1; ALMT9, aluminum-activated malate transporter 9; BAK1, BRI1-associated receptor kinase 1; BLUS1, blue light signaling 1; CPK3/6/23, calcium-dependent protein kinase 3/6/23; HT1, high leaf temperature 1; KAT1/2, potassium channel protein 1/2; OST1, open stomata 1; PHOT2, phototropin 2; PYL9, pyrabactin resistance 1-like 9; QUAC1, quick-activating anion channel 1; SLAC1, slow anion channel-associated 1. White and black bars indicate daytime (12 h) and nighttime (12 h), respectively.

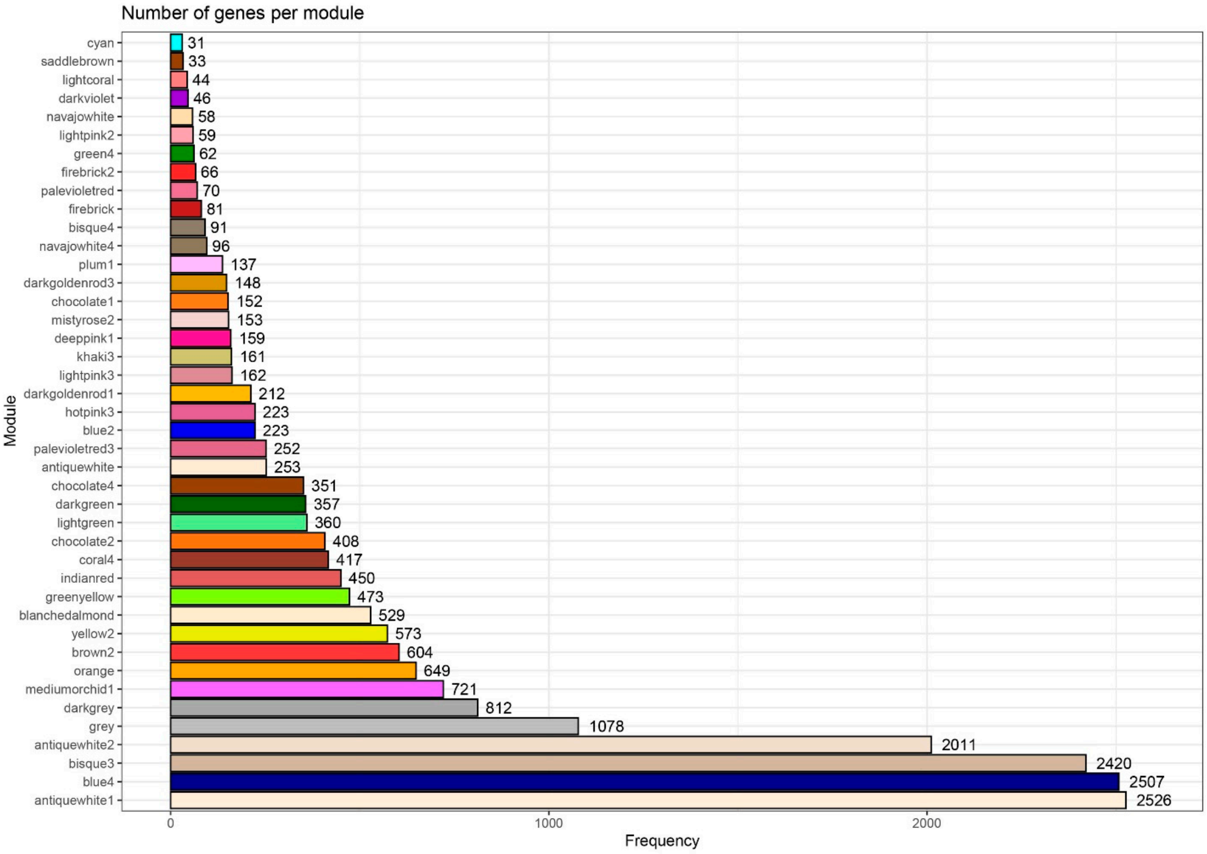

Supplementary Figure S3. Distribution of genes among distinct co-expression modules identified through weighted gene co-expression network analysis (WGCNA) in *Kalanchoe fedtschenkoi*.

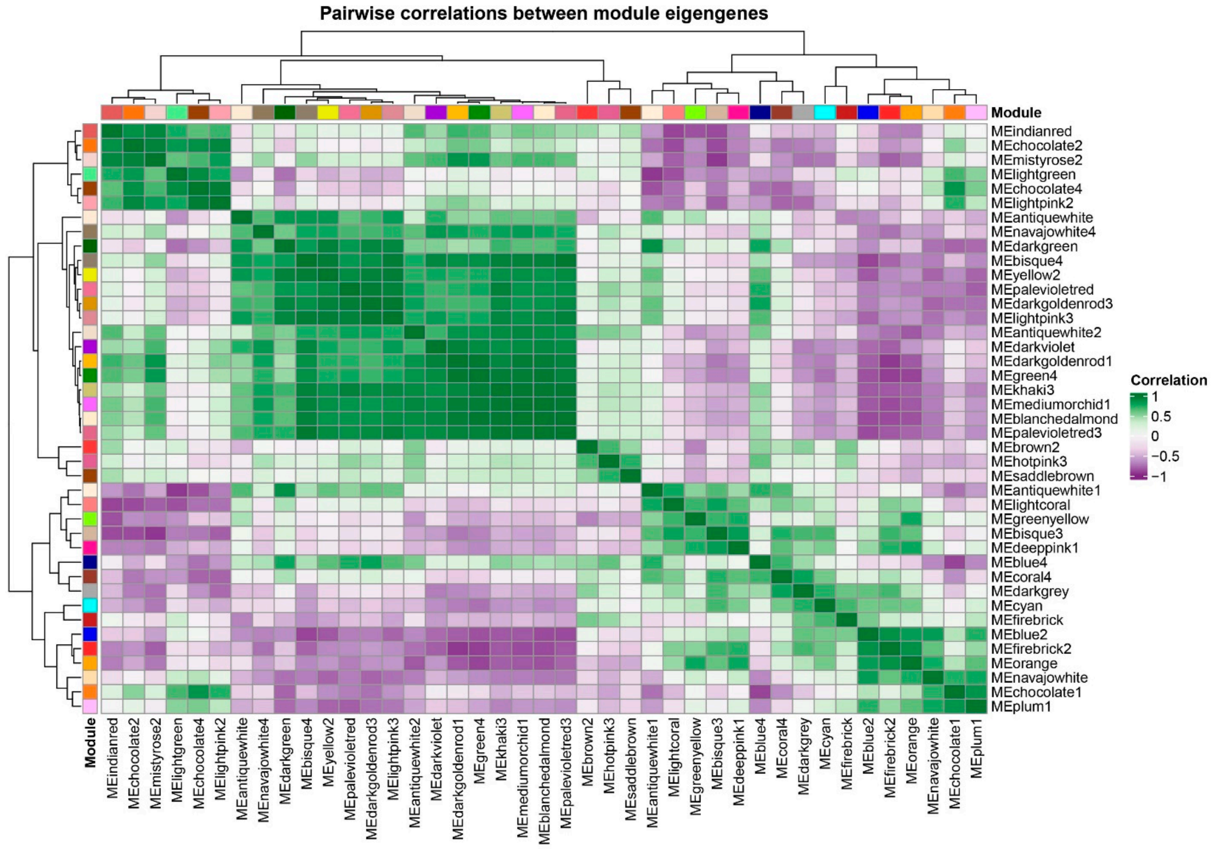

Supplementary Figure S4. Heatmap showing pairwise co-expression correlations and eigengene adjacencies among distinct gene co-expression modules identified through weighted gene co-expression network analysis (WGCNA) in *Kalanchoe fedtschenkoi*.

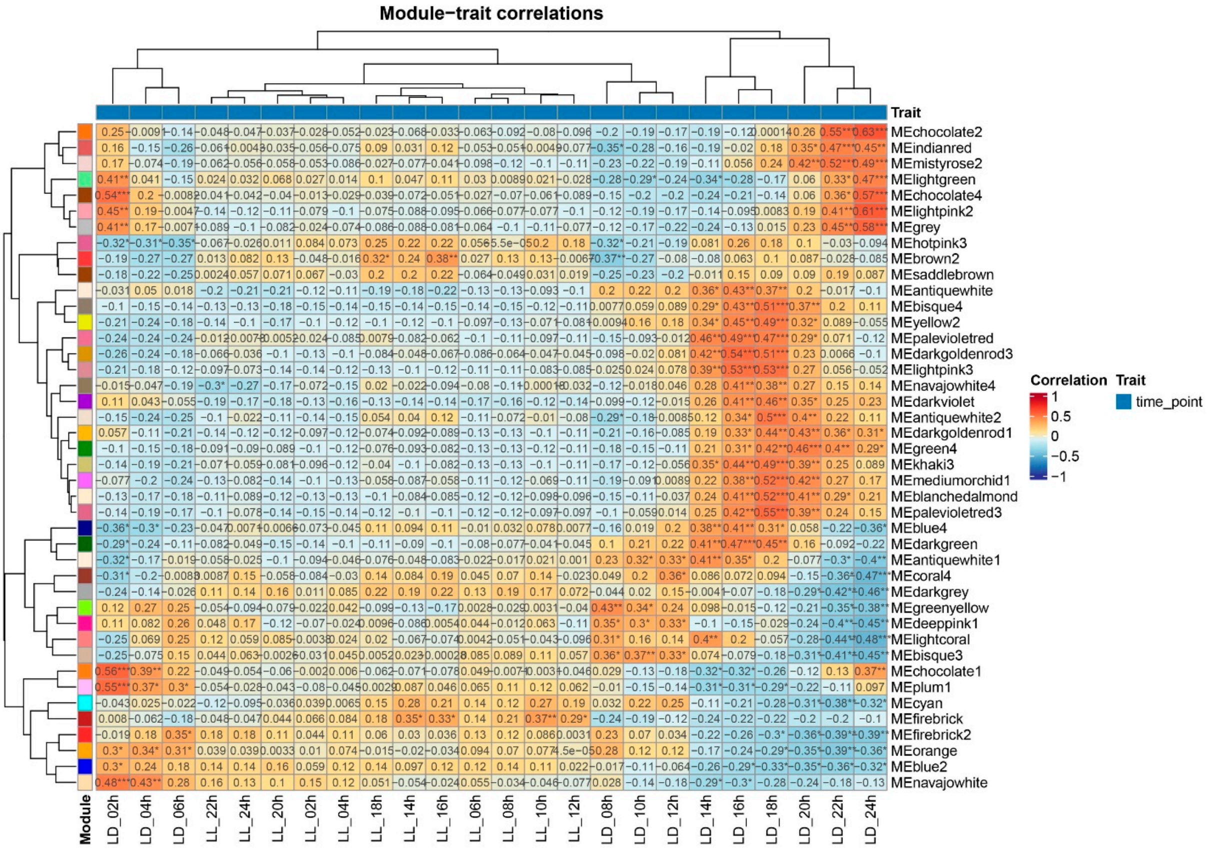

Supplementary Figure S5. Heatmap showing correlations between gene co-expression modules and rhythmic regulation in *Kalanchoe fedtschenkoi*.

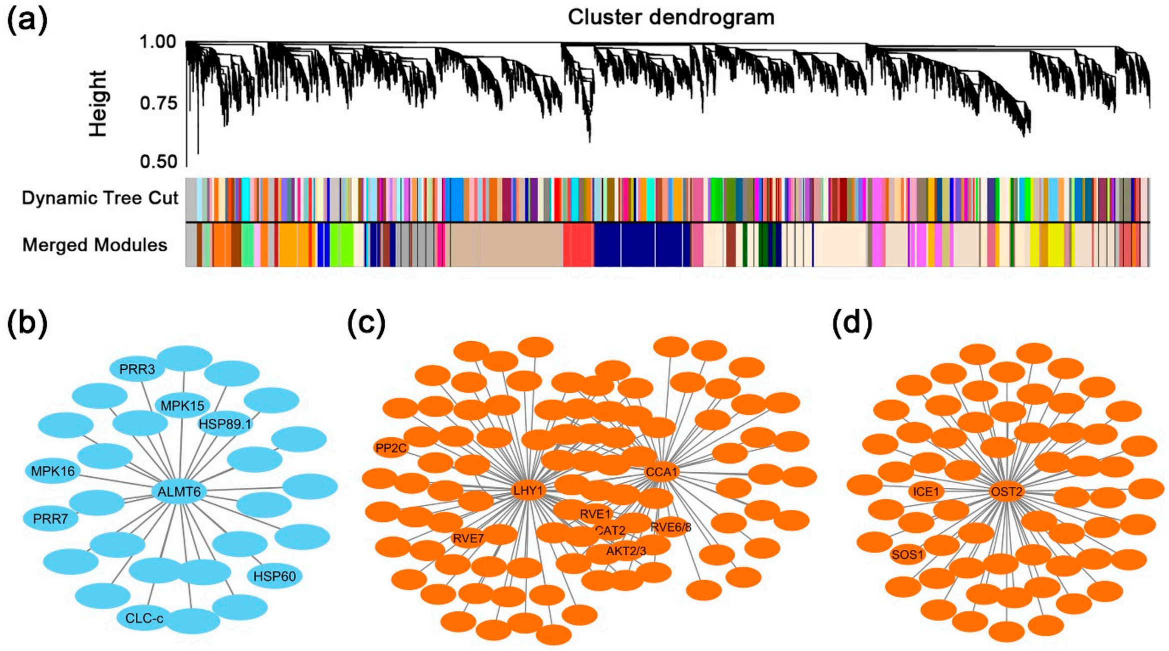

Supplementary Figure S6. Weighted gene co-expression network analysis (WGCNA) of rhythmically expressed genes under LD, LL and LD-LL conditions in *Kalanchoe fedtschenkoi*. **(a).** Cluster dendrogram of rhythmically expressed genes. A total of 42 different modules (MEs) were identified in distinct colors. **(b).** Extracted subnetwork for CAM gene *ALMT6*. *MPK15/16*, mitogen-activated protein kinase 15/16; *HSP60/89.1*, heat shock protein 60/89.1; *PRR3/7*, pseudo-response regulator. **(c).** Extracted subnetwork for *LHY1* and *CCA1*. *RVE1/7/6/8,* REVEILLE transcription factors 1/7/6/8; *AKT2/3*, potassium transport 2/3. **(d).** Extracted subnetwork for *OST2*. *ICE1*, inducer of CBF expression 1; *SOS1*, salt overly sensitive 1.

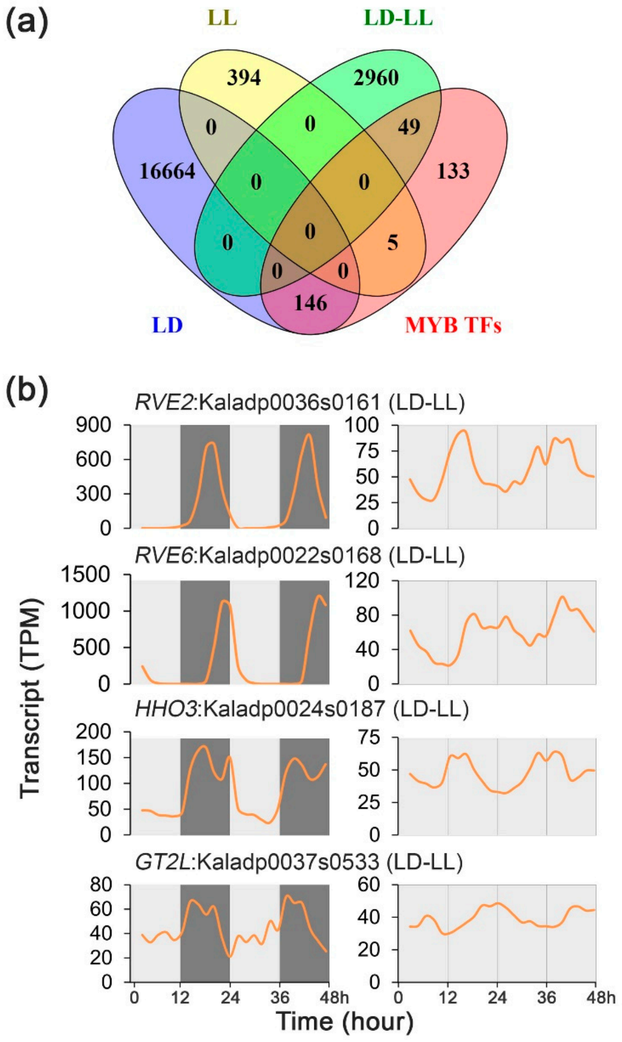

Supplementary Figure S7. Transcript profiles of MYB transcription factor (TF) genes under 48 h time-series light/dark (LD) and constant light (LL) conditions in *Kalanchoe fedtschenkoi*. **(a)**. Venn diagram analysis for category identification of MYB TFs. **(b)**. Expression patterns of selected MYB TF genes under LD and LL conditions. Orange lines represent transcript abundance. White and black bars indicate daytime (12 h) and nighttime (12 h), respectively.

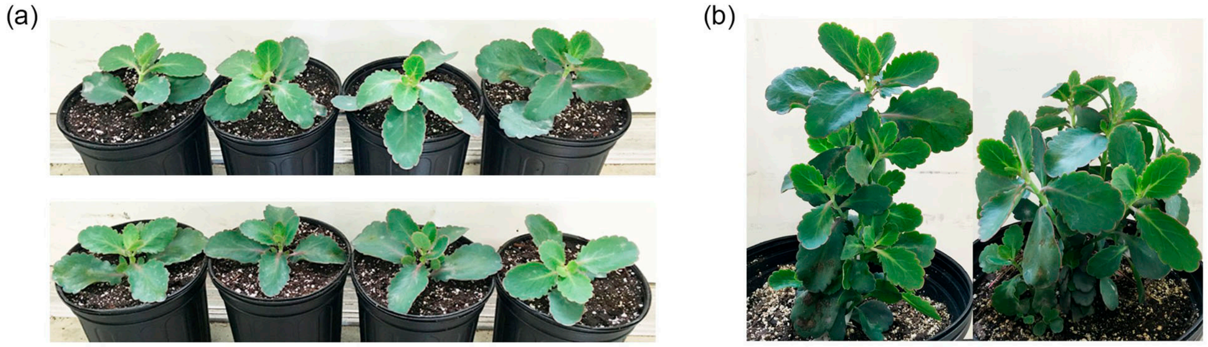

Supplementary Figure S8. Propagation of *Kalanchoe fedtschenkoi* plants. **(a).** Newly propagated plants generated from fresh stem cuttings. **(b).** Four-week-old plants used for subsequent light/dark cycle and constant light treatments.
